# *GM-CSF-miRNA-Jak2/Stat3* Signaling Mediates Chemotherapy-Induced Cancer Cell Stemness in Gastric Cancer

**DOI:** 10.3389/fphar.2022.855351

**Published:** 2022-05-05

**Authors:** Xue Xiang, Hai-zhong Ma, Ya-qiong Chen, Dong-zhi Zhang, Shi-xu Ma, Hong-jing Wang, De-ming Liu, Yuan Yuan, Hui Cai

**Affiliations:** ^1^ Gansu General Surgery Clinical Medical Center, Gansu Provincial Hospital, Lanzhou, China; ^2^ Department of Clinical Medicine, Ningxia Medical University, Yinchuan, China; ^3^ Key Laboratory of Molecular Diagnostics and Precision Medicine for Surgical Oncology in Gansu Province, Gansu Provincial Hospital, Gansu, China; ^4^ NHC Key Laboratory of Diagnosis and Therapy of Gastrointestinal Tumor, Gansu Provincial Hospital, Lanzhou, China

**Keywords:** gastric cancer, cancer stem cells, GM-CSF, miR-877–3p, chemotherapy

## Abstract

Chemotherapy serves as the first choice in clinic to treat advanced gastric cancer. However, emerging evidence indicated the induction of drug resistance and cancer stem cells occasionally by chemotherapy, which seriously limit the therapeutic effects, but the regulatory mechanism remains unclear. Here we treated two human gastric cancer cell lines SGC7901 and BGC823 with 5-Fluorouracil (5-Fu) or Cisplatin (DDP) *in vitro*. The survived cells showed significant increase of drug resistance, cell stemness and cytokine *GM-CSF* expression and secretion. As such, *GM-CSF* was applied to stimulate gastric cancer cells, followed by the subpopulation of *CD133^**+**^* CSC analysis, sphere formation assay and stemness genes expression analysis. As a result, CSCs showed induction by *GM-CSF* treatment. A gastric cancer animal model further indicated that the gastric cancer cells significantly promoted tumor growth after *GM-CSF* treatment *in vivo*. High-throughput miRNA and mRNA sequencing analyses identified a subset of miRNAs and mRNAs under regulation of both 5-Fu and *GM-CSF* in gastric cancer cells, including upregulation of *miR-877-3p* and downregulation of *SOCS2*. Targeted overexpression or knockdown of *miR-877-3p* in gastric cancer cells revealed the oncogenic function of *miR-877-3p* in regulating gastric cancer by suppressing target gene *SOCS2. Jak2/Stat3* signaling pathway, as a downstream target of *SOCS2*, showed activation *in vitro* and *in vivo* after treatment with *miR-877-3p* or *GM-CSF*. Our findings not only revealed a novel mechanism through which chemotherapy induced CSCs in gastric cancer via *GM-CSF-miRNA-Jak2/Stat3* signaling, but also provided an experimental evidence for appropriate dose reduction of adjuvant chemotherapy in treatment of cancer patients.

## Introduction

Gastric cancer (GC), as the fourth leading cause of cancer death all over the world (Sung et al., 2021), shows good clinical outcomes to chemotherapy including 5-Fluoride (5-FU) and Cisplatin (DDP) (Seo et al., 2019). However, chemo-resistance is commonly observed in patients with GC after chemotherapy (Choi et al., 2002). Chemotherapy-induced resistance was reported to be related to the acquisition of stem cell-like properties in cancer cells (Li and Clevers, 2010; Xu et al., 2015). This type of cell is called cancer stem cells (CSCs), which are characterized by self-renewal, differentiation, strong tumor-regenerative ability and resistance to therapy. CSCs are believed to play important roles in tumor invasion, cancer metastasis and cancer recurrence (Alison et al., 2012; Schulenburg et al., 2015). Since the first identification of CSCs in myeloid leukemia in 1997, numerous studies have identified CSCs in multiple types of solid cancer tumors including breast cancer, brain cancer, prostate cancer and GC (Bonnet and Dick, 1997; Al-Hajj et al., 2003; Ricci-Vitiani et al., 2007; Takaishi et al., 2009). Although the regulation of chemo-resistance and cancer relapse by CSCs has been well demonstrated, the molecular mechanisms remain unclear.

Tissue damage and inflammatory response caused by chemotherapy are believed as one of the main causes of chemo-resistance. In addition to kill cancer cells, chemotherapy causes the abundance changes of a variety of inflammatory factors in the microenvironment, affecting the chemotherapeutic outcomes (Edwardson et al., 2019). For example, granulocyte-macrophage colony-stimulating factor (*GM-CSF*), as a monomeric cytokine involving in the immune modulation and hematopoiesis, can be induced by chemotherapy (Hong, 2016; O'Shaughnessy et al., 1994). *GM-CSF* is mostly secreted by activated monocytes, macrophages, T cells, B cells, fibroblasts, mast cells, vascular endothelial cells, and a variety of cancer cells (Shi et al., 2006), regulating proliferation and maturation of immune cells including dendritic cells, granulocytes and macrophages (McLeish et al., 1998; Pei et al., 1999; Ju et al., 2016). Emerging evidence indicates *GM-CSF* acting as a tumor-driver in some cases by promoting tumor growth and progression in multiple cancer types, such as meningiomas, gliomas, skin cancer, head and neck cancer, lung cancer, and so on (Pei et al., 1999; Obermueller et al., 2004; Gutschalk et al., 2006; Uemura et al., 2006; Hong, 2016; Sielska et al., 2020).

MicroRNAs (miRNAs) are a class of highly conserved small non-coding RNA with 18–24 nucleotides in length. Typically, miRNAs bind to the 3′-untranslated region (3′-UTR) of target mRNAs, directing the formation of miRNA-mRNA silencing complexes and leading to degradation or translational inhibition of the targeted mRNAs (Bartel, 2009; Su et al., 2015). MiRNAs play an important role in regulating cancer cell stemness, tumor regeneration, metastasis and chemo-resistance during the development and progression of cancer (Sun et al., 2014; Rupaimoole and Slack, 2017) via targeting various signaling pathways including *Wnt, Akt, Jak/Stat*, et al. (Gomes et al., 2016; Matsui, 2016; Mihanfar et al., 2019). For example, *miR-106a-3p* induced apatinib resistance in gastric cancer cells by targeting the Cytokine signaling (SOCS) system and activating *Jak2/Stat3* signaling (Guo et al., 2019). Activation of the *Jak2/Stat3* signaling promotes cell proliferation and cell stemness in cancer (Yu et al., 2014; Park et al., 2019). SOCS proteins function as negative regulators of cytokine-triggered cell signaling. In gastric cancer, *Jak/Stat* signal pathway is frequently deregulated by the SOCS family and miRNAs (Zhou et al., 2015; Guo et al., 2019).

In the current study, we demonstrated the increased level of *GM-CSF* both inside and outside of the survived gastric cancer cells after treatment with 5-FU or DDP, which was associated with promoted drug resistance and cell stemness. In order to determine the relationship between the increased *GM-CSF* level and promoted cell stemness after chemotherapy in GC, exogenous *GM-CSF* was applied to the culture medium of GC cells, followed by the analysis of *CD133**^+^*** CSC subpopulation, indicating positive regulation of cancer cell stemness by *GM-CSF* stimulation *in vitro*. A GC animal model further demonstrated increased growth of tumors derived from the *GM-CSF*-treated GC cells *in vivo*. To further reveal the regulatory mechanism, high-throughput miRNA and mRNA sequencing analyses were applied to the GC cells before and after chemotherapy or *GM-CSF* treatment. As a result, a subset of miRNAs was identified with deregulation upon treatment with 5-FU or *GM-CSF*, including upregulation of *miR-877-3p* and downregulation of *SOCS2*. Functional assays demonstrated that *miR-877-3p* is capable to promote GC cell proliferation and cell stemness. *SOCS2* was identified as a key direct target gene of *miR-877-3p* in GC, where *miR-877-3p* suppressed the expression of *SOCS2* and promoted cancer cell stemness and chemoresistance subsequently by activating *Jak2/Stat3* signaling. The current study is the first to demonstrate a mechanism through which *GM-CSF-miRNA-Jak/Stat* signaling mediates chemotherapy-induced cell stemness and drug resistance in gastric cancer.

## Materials and Methods

**Animals.** Six-week-old immune-deficient female nude mice were purchased from the SiPeiFu Animal Company (Beijing, China) for *in vivo* assays. 2×10^6^ SGC7901 cells with or without *GM-CSF* stimulation were transplanted per mouse by subcutaneous injection to establish the animal model with gastric cancer. All animal studies were performed following the relevant guidelines, regulations and protocols approved by our Institutional Animal Care and Use Committee.

**Cells.** Human gastric cancer cell lines SGC7901 and BGC823 were purchased from the cell bank of the Chinese Academy of Sciences at Shanghai, China, maintained in our lab, and cultured in Dulbecco’s Modified Eagle’s Medium (DMEM) with 1% penicillin-streptomycin and 10% fetal bovine serum (Gibco, United States). All of these cells were cultured at 37°C with 5% CO_2_ in a humidified environment.

**RNA Extraction, miRNA and mRNA sequencing, Bioinformatics analysis.** Total RNA was extracted using Trizol reagent (Invitrogen, Thermo Fisher Scientific) following the manufacturer’s instructions. The quantity of the total RNA was accessed by NanoDrop One spectrophotometer (Thermo Fisher Scientific), and the integrity of the RNA was assessed by Bioanalyzer 2,100 (Agilent, CA, United States) with RIN number >7.0, and confirmed by electrophoresis with denaturing agarose gel. After quality check, approximately 1 μg of total RNA was used to prepare small RNA library according to protocol of TruSeq Small RNA Sample Prep Kits (Illumina, San Diego, United States), and approximately 1 μg of total RNA was used for mRNA library. In two libraries, we performed the single-end sequencing (1 × 50 bp) on an Illumina Hiseq2500 and paired-end sequencing (2 × 150 bp) on an illumine Novaseq™ 6000 LC-Bio Technology Company, Ltd., (Hangzhou, China) following the vendor’s recommended protocol. Differentially expressed miRNAs based on normalized deep-sequencing counts were analyzed using Student’s t-test. The screening criteria were a fold change >−2 and *p* < 0.01. The differentially expressed mRNAs were selected with log2 (fold change) > 1 or log2 (fold change) <−1 and with statistical significance (*p*-value < 0.05) by the edgeR package. After quality control, bioinformatics analyses (Heatmaps and Venn diagram) were performed with the online OmicStudio tools at http://www.omicstudio.cn/tool.

**Plasmids, oligos, and transfection.** *miR-877-3p* mimics, *anti-miR-877-3p* inhibitors, and corresponding negative controls were synthesized by RiboBio Co., Ltd. (Guangzhou, China). Firefly luciferase reporter plasmids carrying either wild type or mutated *SOCS2* 3′UTR were constructed by Genomeditech company (Shanghai, China). Oligo transfection was performed using lipofectamine 2000 (Invitrogen, United States) following the manufacturer’s instructions. A final concentration of 30 nM of miRNA mimic or negative control was used in all *in vitro* assays.

**First strand cDNA preparation and Real-Time PCR.** Total RNAs were extracted by using Trizol reagent (Invitrogen, Thermo Fisher Scientific). The method of adding a poly A tail to small RNAs was used for reverse transcription of miRNAs. Prime script™ RT Reagent kit with gDNA Eraser (Takara, Japan) was used for reverse transcription of mRNAs. Power Up SYBR Green Master Mix (Applied Biosystem, Thermo Fisher Scientific) and Applied Biosystems QuantStudio 6 (Applied Biosystem, Thermo Fisher Scientific) were used for real-time PCR assays. GAPDH and 5s rRNA were used for mRNA and miRNA normalization. GAPDH forward: 5’-GGAGCGAGATCCCTCCAAAAT-3’; reverse: 5’-GGCTGTTGTCATACTTCTCATGG-3’; 5s forward: 5’-AGTACTTGGATGGGAGACCG-3’; miR-877-3p forward: 5’-UCCUCUUCUCCCUCCUCCCAG-3’.

**Quantitative analysis of *GM-CSF*.** Secreted *GM-CSF* in the supernatant of SGC7901 or BGC823 cells before or after treatment with 5-FU or DDP was quantified using sandwich ELISA following the manufacturer’s instructions (Multi Sciences, Hangzhou, China).

**Western Blot.** Cells were lysed in RIPA buffer (Beyotime, China), and protein concentration was measured using a BCA Assay Kit (Beyotime, China). 50μg protein lysates were prepared and resolved by 8–12.5% sodium dodecyl sulfate–polyacrylamide gel electrophoresis (SDS/PAGE) and transferred onto an Immuno-Blot Polyvinylidene difluoride (PVDF) membrane (Millipore, United States). The membranes were then blocked with 5% non-fat milk in TBST for 1 h at room temperature and subsequently incubated with the primary antibodies in 1:1,000 dilution overnight at 4°C. After washing with TBST three times, then the membrane was incubated with the secondary antibody for 1 h at room temperature. Protein bands were visualized using the Minichemi chemiluminescence Imaging System (Beijing Sage Creation Science Co., Ltd., China). The following antibodies were used for Western blot: anti-*SOCS2* (2779T, Cell Signaling Technology), anti-*JAK2* (3230T, Cell Signaling Technology), anti-*p-JAK2* (4406T, Cell Signaling Technology), anti-STAT3 (9139T, Cell Signaling Technology), anti-*p-STAT3* (9145T, Cell Signaling Technology), anti-*OCT4* (2750S, Cell Signaling Technology), anti-*NANOG* (4903S, Cell Signaling Technology), anti-*GAPDH* (sc-47724, Santa Cruz), anti-*KLF4* (sc-393462, Santa Cruz), anti-*h-TERT* (sc-377511, Santa Cruz), anti-*GM-CSF* (sc-32753, Santa Cruz) and anti-*β-tubulin* (ab18207, Abcam). Secondary antibodies (1:10,000) were HRP-linked anti-rabbit IgG (7074S, Cell Signaling Technology) and HRP-linked anti-mouse IgG (7076S, Cell Signaling Technology).

**Cell proliferation assay.** For proliferation assay, 3,000 cells per well were seeded into 96-well culture plates in triplicate. and incubated for 2 days at 37°C in a humidified incubator with 5% CO_2_. Every 24 h interval, each well was added with 10 μL CCK-8 solution (SB-CCK8, Sharebio, Shanghai, China), then cultured for 3 h at cell culturing condition followed by measurement of OD value at 450 nm wavelength (SpectraMax M5, MolecularDevices, United States).

**Colony formation assay.** Cancer cells were plated into a 6-well plate at a density of 2,000 cells/well, and after 7-14 days culture until visible colonies were formed. Then, colonies were washed with PBS and fixed with 4% paraformaldehyde. Finally, the visible colonies were stained with 0.5% crystal violet for 20 min. All experiments have three repetitions.

**Sphere formation assay.** After GC cells were transfected with miRNA-877-3p (mimic, negative control and inhibitor) for 24h, 2,000 GC cells per well were seeded into a 6-well ultra-low attachment cell culture plate (Corning, United States), and cultured with 20 ng/ml of bFGF (R&D Systems, United States), 20 ng/mL EGF (Sigma, United States), and 1×B27 supplement (Invitrogen, United States) in stem cell medium DMEM/F12. The number and sizes of tumorsphere in each well were determined after incubation for 10 days.

**Luciferase reporter assay.** pGL-3 luciferase reporter plasmids carrying either wild type or mutated SOCS2 3’UTR and Renilla luciferase plasmid (pRL-TK) were co-transfected into 293T cells with miR-877-3p mimic or negative control in a 24-well plate. After 18-h transfection. Luciferase activities were determined with the Dual-Luciferase Reporter Assay kit (Promega, USA).

**Statistical analysis.** Quantitative data are expressed as mean ±SEM unless otherwise stated. Statistical significance was determined using Student’s t-test followed by least-significant difference (LSD). The data were considered to be significant when the P < 0.05.

## Results

**FIGURE 1:**
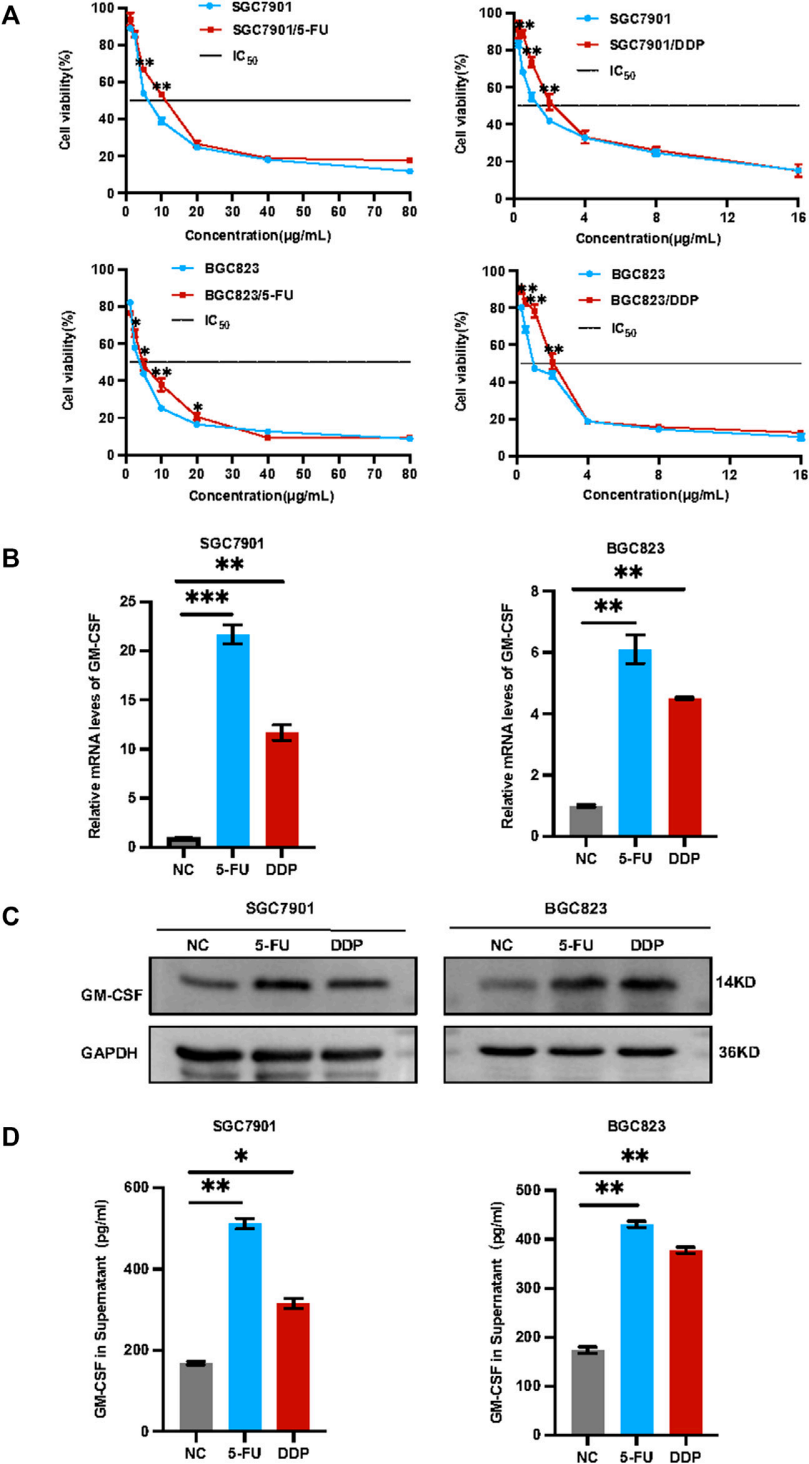
Induction of drug resistance and *GM-CSF* expression/secretion in the survived gastric cancer cells after chemotherapy. **(A)**: IC_50_ analysis of the survived SGC7901 and BGC823 cells after treatment with 5-FU or DDP for 72 h. **(B)**: QRT-PCR analysis of the *GM-CSF* mRNA levels in the survived SGC7901 and BGC823 cells. **(C)**: Western blot analysis of the GM-CSF protein levels in the survived SGC7901 and BGC823 cells. **(D)**: ELISA analysis of the *GM-CSF* levels in supernatants of the survived SGC7901 and BGC823 cells. Data are presented as the mean ± SEM (*N* = 3). *p < 0.05, **p < 0.01, ***p < 0.001.

**Induction of drug resistance and *GM-CSF* expression/secretion by chemotherapy in gastric cancer.** In view of observation GM-CSF is overexpressed in tumor cells after radiotherapy and induced tumor migration (Vilalta et al., 2014; Vilalta et al., 2018). GM-CSF combined with chemoradiation could trigger abscopal effect (Benna et al., 2020). Highly expressed granulocyte colony-stimulating factor (G-CSF) and granulocyte colony-stimulating factor receptor (G-CSFR) leads to poor survival in gastric cancer (Fan et al., 2018). Tumor-derived GM-CSF promotes gastrointestinal tumorigenesis (Wang et al., 2014), we herein applied *in vitro* and *in vivo* assays to validate the phenotypes and determine the regulatory mechanism. Human gastric cancer cells SGC7901 and BGC823 were treated with a low concentration of 5-FU or DDP for 72 h *in vitro*. Survived cells were collected for further analysis including IC_50_, cell stemness, as well as *GM-CSF* levels. As shown in Figure 1A, both survived SGC7901 and BGC823 cells showed increased IC_50_ and drug resistance, associated with increased *GM-CSF* levels at both mRNA and protein levels in cells (Figures 1B,C) and in secretion in the supernatant (Figure 1D).

**FIGURE 2:**
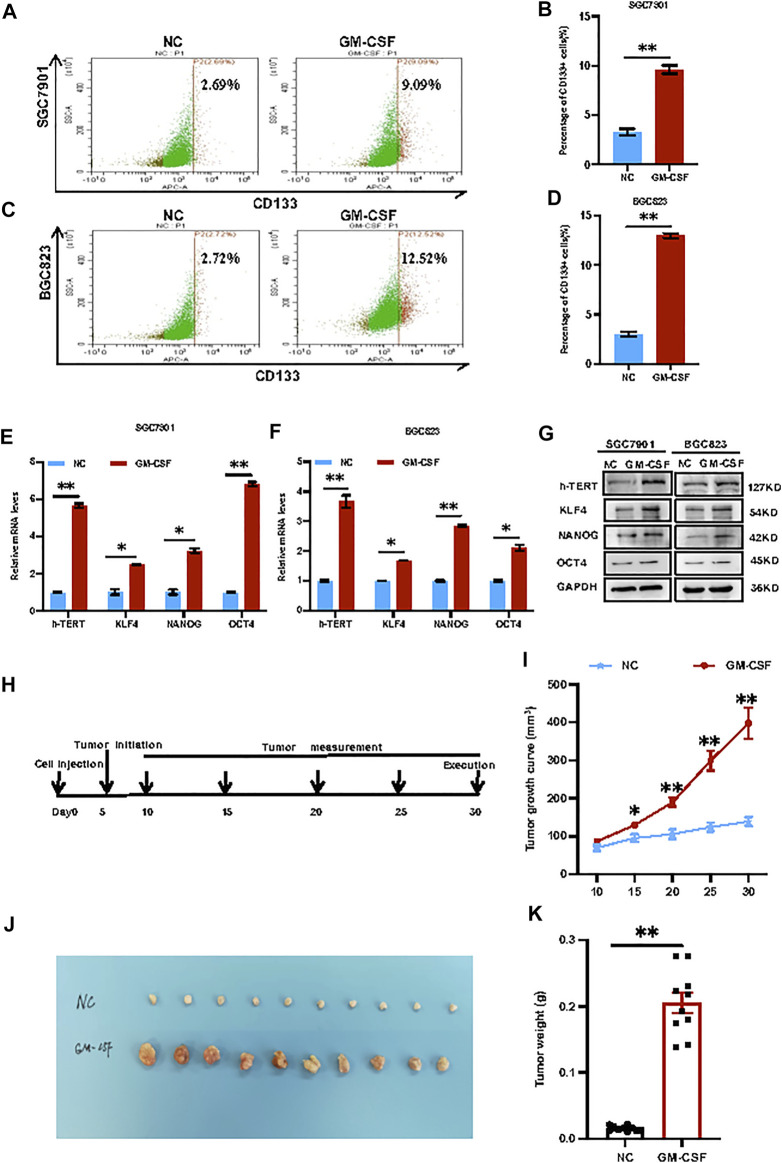
*GM-CSF* treatment promoted cancer cell stemness *in vitro* and tumorigenesis *in vivo*. **(A,C)**: Flow cytometry analysis of *CD133* ^***+***^ CSCs in SGC7901 **(A)** and BGC823 **(C)** cells before and after stimulation with exogenous GM-CSF in the cell culture medium. B and D: Quantitative analysis of A **(B)** and C **(D)**. **(E,F)**: QRT-PCR analysis of the stemness genes expression (*h-Tert*, *Klf4*, *Nanog* and *Oct4*) in SGC7901 **(E)** and BGC823 **(F)** cells with or without treatment with *GM-CSF*. **(G)**: Western blot analysis of the stemness genes expression in SGC7901 and BGC823 cells with or without treatment with *GM-CSF*. **(H)**: A gastric cancer xenograft model by transplantation of SGC7901 cells with or without stimulation with *GM-CSF* into nude mice (*n* = 10 in each group). **(I)**: Tumor growth curves in **(H)**. **(J)**: Tumor images in **(H)**. **(K)**: Tumor weight in **(H)**. Data are presented as the mean ± SEM (*N* = 3 for *in vitro* assays, and *N* = 10 for *in vivo* assay). *p < 0.05, **p < 0.01, ***p < 0.001.

***GM-CSF* treatment promoted cancer cell stemness *in vitro* and tumorigenesis *in vivo*.** SGC7901 and BGC823 cells were stimulated with exogenous GM-CSF by adding into the cell culture medium, followed by the *CD133* ^+^ CSC subpopulation analysis. As a result, The *CD133* ^+^ CSC subpopulation increased from 2.69% to 9.09% in SGC7901 cells (Figures 2A,B), and from 2.72% to 12.52.% in BGC823 cells (Figures 2C,D) after stimulation, respectively. In addition, a group of well-defined stemness genes including *h-Tert, Klf4, Nanog* and *Oct4* was examined by quantitative RT-PCR and western blot analyses in the 2 GC cell lines before or after treatment with *GM-CSF*. In consistent with the results in Figures 2A–D, these stemness genes showed induction in expression at both mRNA and protein levels by *GM-CSF* stimulation (Figures 2E–G).

In order to further determine the effects of *GM-CSF* on tumorigenesis *in vivo*, a gastric cancer xenograft model was established by transplantation of SGC7901 cells with or without stimulation with *GM-CSF* into immunodeficient female nude mice through via subcutaneous injection, followed by continuous tracking of the tumor growth (Figure 2H). The tumor growth curves (Figure 2I), tumor images (Figure 2J) and tumor weight (Figure 2K) indicated significant promotion of tumor growth by *GM-CSF* stimulation.

**FIGURE 3:**
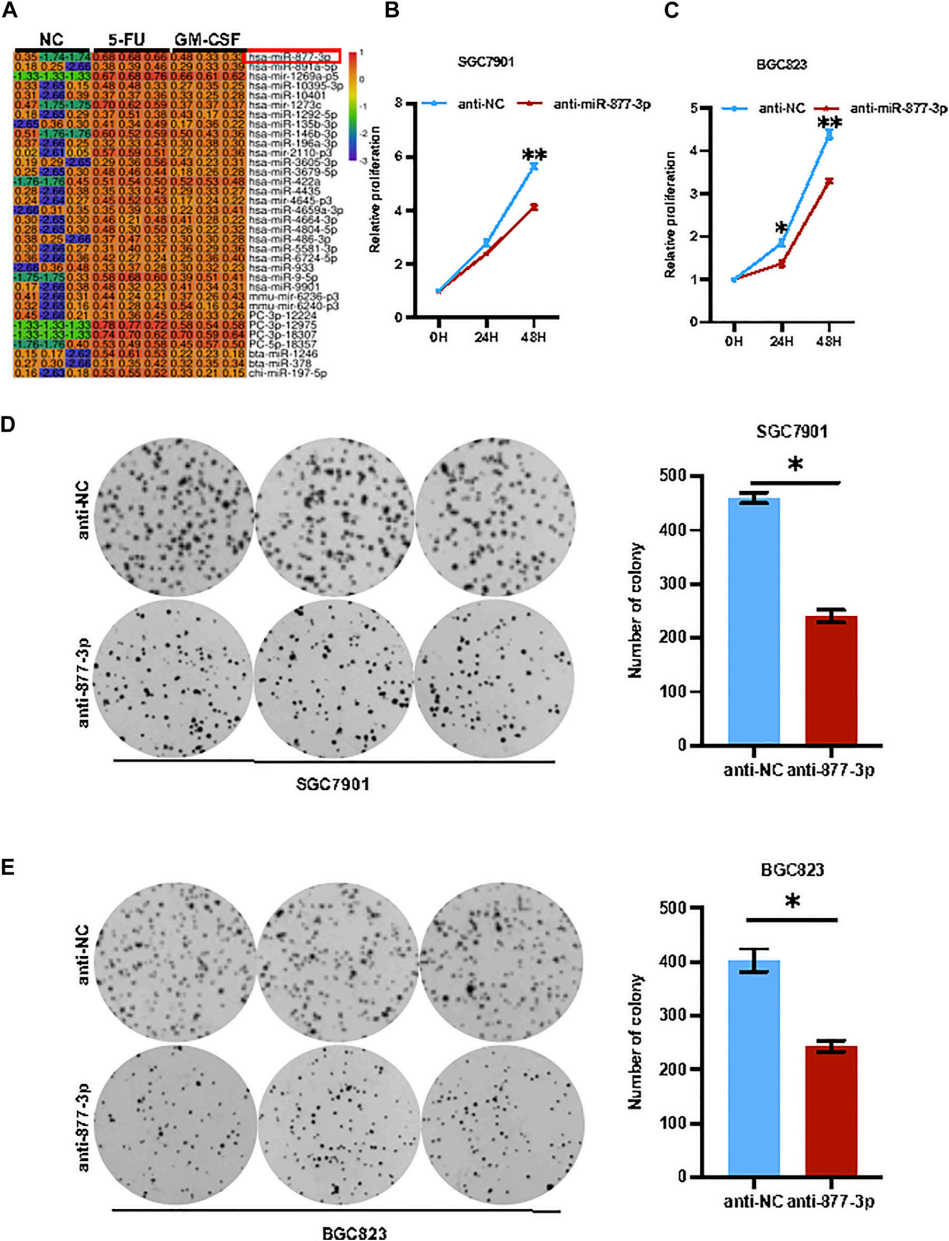
Knockdown of *miR-877-3p* suppressed gastric cancer cell proliferation. **(A)**: High-throughput miRNA sequencing analyses on SGC7901 cells with or without stimulation with 5-FU or *GM-CSF* identified a group of deregulated miRNAs, including *miR-877-3p*. **(B,C)**: Knockdown of *miR-877-3p* in SGC7901 **(B)** and BGC823 **(C)** cells suppressed cell proliferation assayed by CCK8. **(D,E)**: Knockdown of *miR-877-3p* in SGC7901 **(D)** and BGC823 **(E)** cells suppressed the cellular colony formation. Data are presented as the mean ± SEM (*N* = 3). *p < 0.05, **p < 0.01, ***p < 0.001.

**miR-877-3p mediated chemotherapy and *GM-CSF* induced tumor progression in gastric cancer.** To identify the key genes regulating chemotherapy-induced or GM-CSF-induced tumor progression in gastric cancer, SGC7901 cells with or without stimulation with 5-FU or *GM-CSF* were applied for a high-throughput miRNA sequencing analysis. As a result, a subset of miRNAs was identified with a differential expression upon treatment with 5-FU and *GM-CSF*, respectively (Figure 3A). Some miRNAs, such as *miR-9-5p, miR-196a and miR-422a,* have been well documented to regulate tumorigenesis and cancer stem cells in gastric cancer (Pan et al., 2017; He et al., 2018; Liu et al., 2020), while the function of *miR-877-3p* remains unclear in GC. As shown in Supplementary Figure S1, quantitative real-time PCR analysis validated of miR-877-3p overexpression in both SGC7901 and BGC823 cells were treated with 5-FU and DDP respectively. Therefore, we focused on *miR-877-3p* to determine the relationship between upregulation of *miR-877-3p* and chemotherapy-induced drug resistance and cell stemness. Overexpression or knockdown of *miR-877-3p* was applied to gastric cancer cells (Supplementary Figures S2, S3), followed by CCK8 cell proliferation and colony formation assay. As shown in Figures 3B–E, knockdown of *miR-877-3p* suppressed cell proliferation and colony formation in both SGC7901 and BGC823 cells, respectively. Whereas overexpression of *miR-877-3p* dramatically increased cell proliferation and colony formation in both SGC7901 and BGC823 cells (Supplementary Figure S6).

**FIGURE 4:**
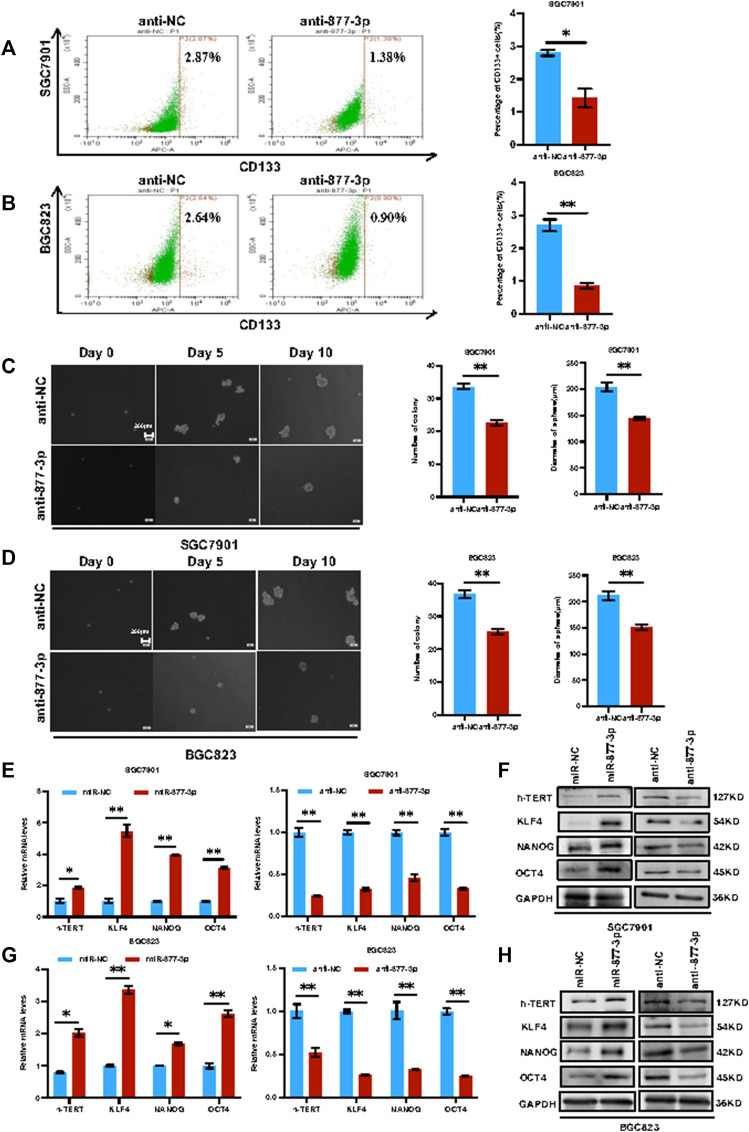
Knockdown of *miR-877-3p* suppressed gastric cancer cell stemness. **(A,B)**: Knockdown of *miR-877-3p* in SGC7901 and BGC823 cells suppressed the subpopulation of CD133^**+**^ CSCs. **(C,D)**: Knockdown of *miR-877-3p* in SGC7901 and BGC823 cells suppressed the sphere formation ability in the serum-free culture condition. **(E,F)**: QRT-PCR **(E)** and western blot **(F)** analyses of the stemness genes including *h-Tert*, *Klf4*, *Nanog* and *Oct4* in SGC7901 cells with or without overexpression or knockdown of *miR-877-3p*. **(G,H)**: QRT-PCR **(G)** and western blot **(H)** analyses of the stemness genes in BGC823 cells with or without overexpression or knockdown of *miR-877-3p*. Data are presented as the mean ± SEM (*N* = 3). *p < 0.05, **p < 0.01, ***p < 0.001.

**Overexpression of *miR-877-3p* promoted the cell stemness in both SGC7901 and BGC823 cells.** After overexpression or knockdown of *miR-877-3p* in both SGC7901 and BGC823 cells, the changes of the CD133^+^ CSC percentage were determined by flow cytometry analysis. As shown in Figure 4A, knockdown of *miR-877-3p* in SGC7901 cells decreased CD133^+^ CSC subpopulation. Similar results were obtained from BGC823cells (Figure 4B). In addition, As shown in Figures 4C,D, sphere formation assays were performed to further determine the stemness changes after knockdown of *miR-877-3*p in both SGC7901 and BGC823 cells. Quantitative analysis indicated that knockdown of *miR-877-3p* decreased both sphere number and sphere size. Whereas overexpression of *miR-877-3p* dramatically increased CD133^+^ CSC subpopulation and sphere formation in both SGC7901 and BGC823 cells (Supplementary Figure S7). Moreover, a group of well-defined stemness genes including *h-Tert, Klf4, Nanog* and *Oct4* was examined in both SGC7901 and BGC823 cells by quantitative RT-PCR and western blot analyses. The results showed that overexpression or knockdown of *miR-877-3p* remarkably increased or decreased the expression of *h-Tert, Klf4, Nanog* and *Oct4* at both mRNA (Figures 4E,G) and protein levels (Figures 4F, 4H).

**FIGURE 5:**
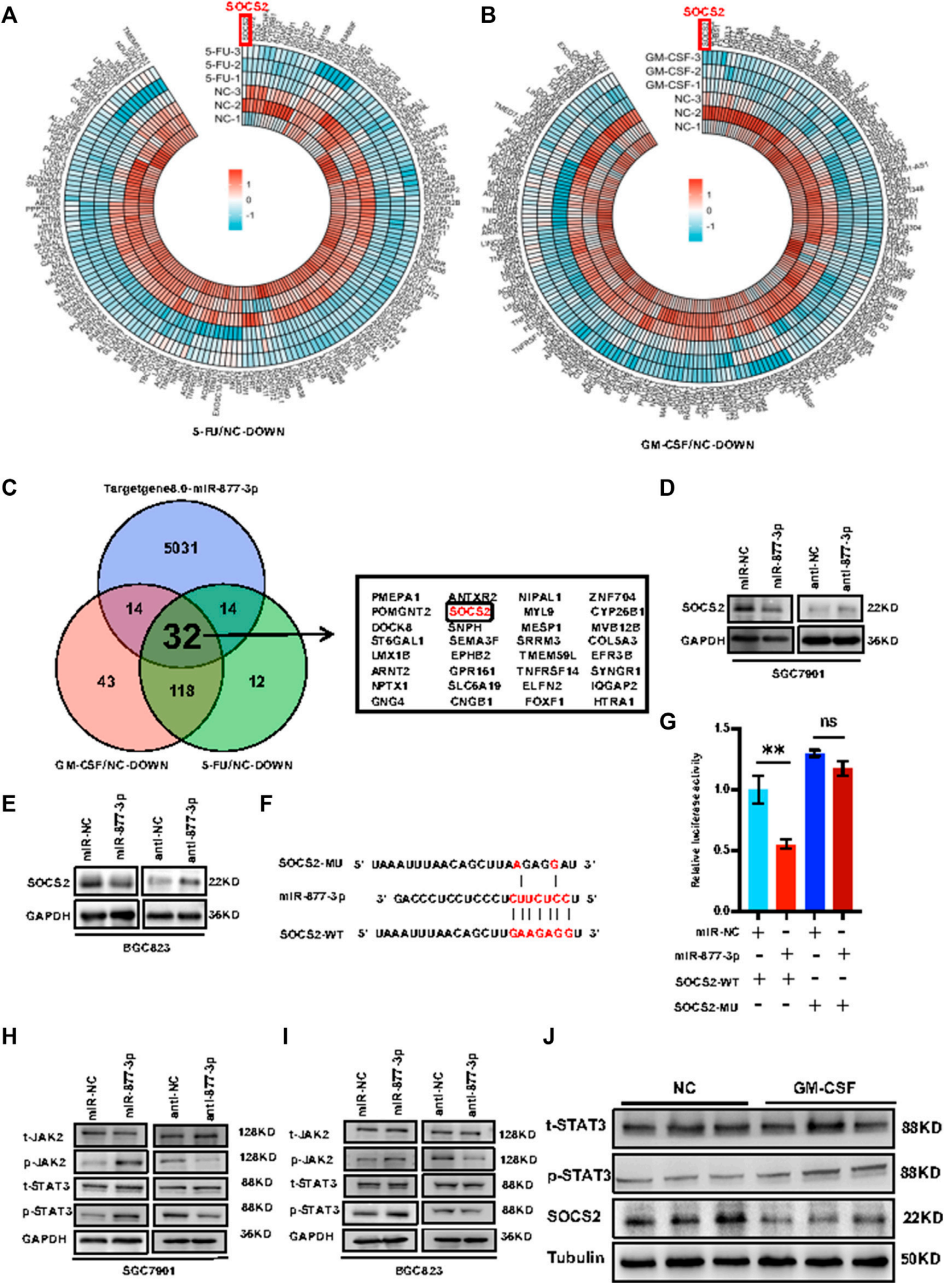
*miR-877-3p* activated *Jak2/Stat3* signaling through targeting *SOCS2* in gastric cancer. **(A,B)**: RNA-seq analysis of the SGC7901 cells with or without stimulation with 5-FU **(A)** or *GM-CSF* **(B)** identified a list of differentially expressed downregulated genes. **(C)**: 32 genes were overlapped from the 176 downregulated genes by 5-FU treatment, 207 downregulated genes by *GM-CSF* treatment, and 5,091 potential target genes of *miR-877-3p* predicted by TargetScan Human8.0, including *SOCS2*. **(D,E)**: Western blot demonstrated inhibition of *SOCS2* by *miR-877-3p* overexpression and promotion of SOCS2 by *miR-877-3p* knockdown in both SGC7901 and BGC823 cells. **(F)**: Sequence alignment of wide type (WT) or *miR-877-3p*-binding sites-mutated (MU) 3′UTR of *SOCS2*. **(G)**: luciferase reporter assay demonstrated inhibition of WT-*SOCS2*-3′UTR by *miR-877-3p*, but not MU-*SOCS2*-3′UTR. **(H,I)**: Western blot demonstrated positive or negative regulation of *p-Jak2* and *p-Stat3* by overexpression or knockdown of *miR-877-3p* in both SGC7901 and BGC823 cells. **(J)**: Western blot demonstrated downregulation of *SOCS2* and activation of *Jak2/Stat3* signaling by *GM-CSF* treatment in the tumors from the mice model. Data are presented as the mean ± SEM (*N* = 3). *p < 0.05, **p < 0.01, ***p < 0.001.

***miR-877-3p* activated *Jak2/Stat3* signaling through targeting *SOCS2*.** In order to determine the molecular mechanism(s) by which *miR-877-3p* promotes gastric cancer development and progression, RNA-seq was applied to the SGC7901 cells with or without stimulation with 5-FU or *GM-CSF*, deriving 176 downregulated genes by 5-FU treatment and 207 downregulated genes by *GM-CSF* treatment (Figures 5A,B). Bioinformatic analysis was using TargetScan Human8.0 (http://www.targetscan.org/vert_80/) predicted 5,091 potential target genes of *miR-877-3p*. From these three groups of genes, 32 genes were overlapped including *SOCS2* (Figure 5C). Quantitative real-time PCR analysis validated downregulation of *SOCS2* at the mRNA levels by overexpression of *miR-877-3p* in both SGC7901 and BGC823 cells (Supplementary Figure S4). Upregulation of *SOCS2* was shown after knockdown of miR-877–3p in both SGC7901 and BGC823 cells (Supplementary Figure S5). Western blot analysis further demonstrated downregulation or upregulation of *SOCS2* at the protein levels by overexpression or knockdown of *miR-877-3p* in both SGC7901 and BGC823 cells (Figures 5D,E). In order to demonstrate the direct interaction between *SOCS2* and *miR-877-3p*, luciferase (Luc) reporter constructs carrying either wide type (WT) or *miR-877-3p*-binding sites-mutated (MU) 3′UTR of *SOCS2* were co-transfected with *miR-877-3p* mimics into 293T cells (Figure 5G). As a result, WT-*SOCS2*-Luc was inhibited by *miR-877-3p*, but MU-*SOCS2*-Luc was not, supporting the target interaction between *SOCS2* 3′-UTR and *miR-877-3p* via sequence complementarity (Figures 5F,G). In view of the well-defined tumor-suppressing function of *SOCS2* by inhibiting *Jak2/Stat3* signaling (Uen et al., 2018; Ren et al., 2019), we detected the effects of *miR-877-3p* on *Jak2/Stat3* signaling in GC. As shown in Figures 5H,I, *p-Stat3* and *p-Jak2* were induced by overexpression of *miR-877-3p*, and suppressed by knockdown of *miR-877-3p* in both SGC7901 and BGC823 cells, which was further validated by western blot analysis on the tumor samples derived from the mouse model (Figure 5J).

**FIGURE 6:**
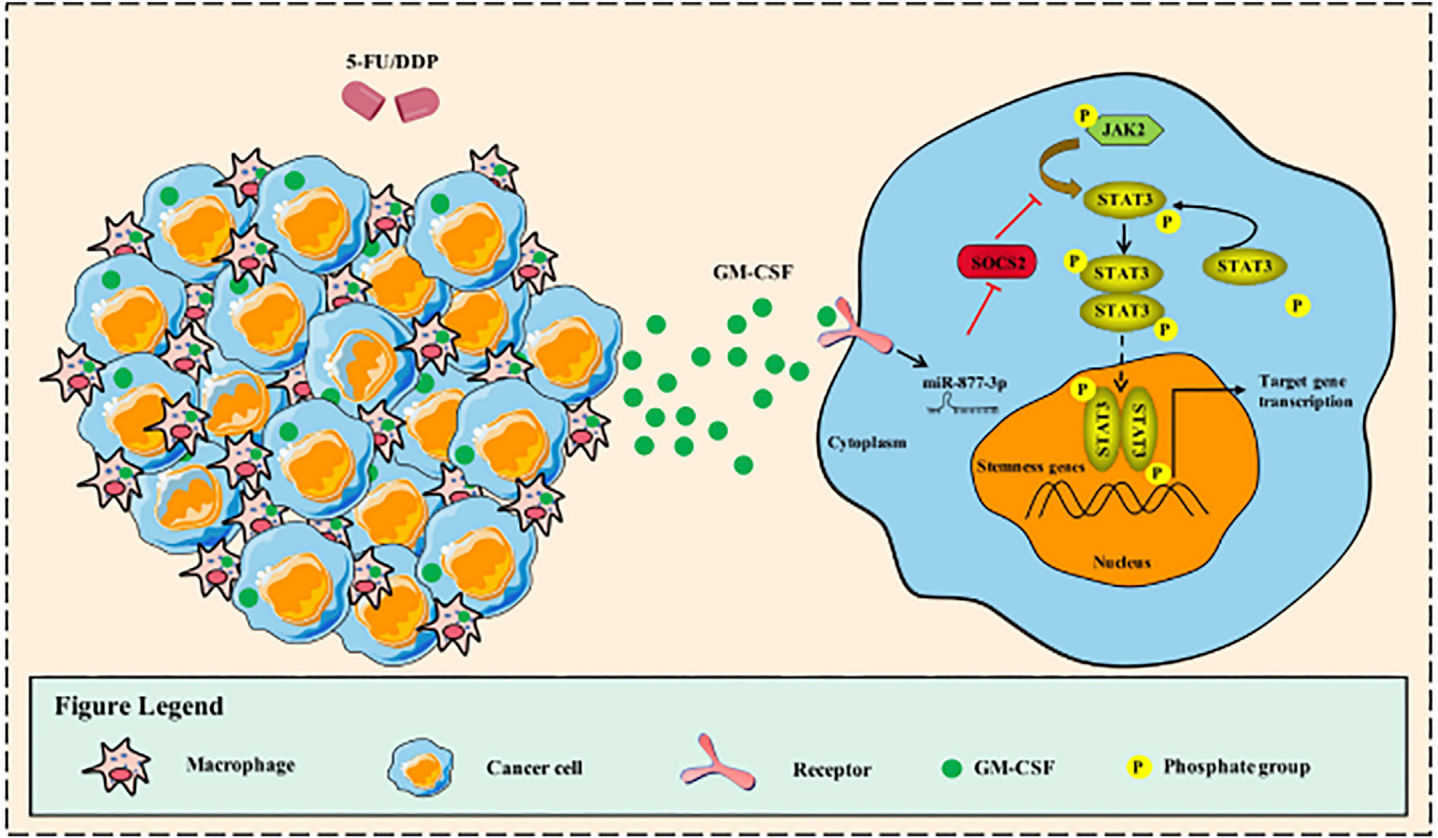
Working Model. Schematic representation of the mechanism through which *GM-CSF* increased the expression of *miR-877-3p* in gastric cancer cells, which suppressed the expression of *SOCS2* as a target gene. *SOCS2*, as a suppressor gene of *Jak2/Stat3* signaling, mediated the chemotherapy-induced cancer cell stemness and drug resistance.

## Discussion

Since *GM-CSF* is able to induce pluripotent stem cells to differentiate into mature granulocytes, macrophages and T cells in bone marrow, it has been used in clinic to protect cancer patients against chemotherapy- or radiotherapy-induced neutropenia. However, emerging evidence found that application of *GM-CSF* therapy occasionally promoted tumor progression (Uemura et al., 2004; Metcalf, 2010), indicating complexity of the *GM-CSF*-based cancer therapy. Herein, we experimentally demonstrated a mechanism through which chemotherapy or *GM-CSF*-based therapy of gastric cancer may induce cancer cell stemness and drug resistance.

Activation of *Jak/Stat3* signaling pathway plays a critical role in promoting tumorigenesis, epithelial and mesenchymal transition (EMT), chemo-resistance, and cancer cell stemness (Jin, 2020). In gastric cancer, overexpression of *p-Stat3* increased sphere formation from *CD44* ^***+***^ CSCs (Hajimoradi et al., 2016). In the current study, we are the first to identify *miR-877-3p* with upregulation in the chemo survived gastric cancer cells, which was mediated by *GM-CSF* induction but in turn suppressed *SOCS2* and activated *Jak/Stat3* signaling. This is in consistence with the literature about the oncogenic function of miR-877-3p in Pancreatic Cancer by interacting with STARD13 (Xu and Zheng, 2020). In addition to *Jak/Stat3*, *PI3k/Akt* and *Erk* signaling pathways have been reported to have interacted with *GM-CSF* in regulating tumor cell proliferation and migration (Kawaguchi et al., 2004; Carlson et al., 2011). Although we did not analyze in the current study whether these two pathways are involved in regulating the *GM-CSF*-induced cell stemness and drug resistance, our high-throughput RNA sequencing data analyses suggested activation of *PI3k/Akt* signaling after *GM-CSF* treatment in gastric cancer.

In conclusion, CSCs are believed to be the main source of cancer initiation, relapse, and drug resistance. Therapeutic strategies targeting CSCs hold great promise in the fight against cancer. The current study demonstrated a novel mechanism regulating chemotherapy-induced CSCs and drug resistance in gastric cancer.

## Data Availability

The datasets presented in this study can be found in online repositories. The names of the repository/repositories and accession number(s) can be found below: National Center for Biotechnology Information (NCBI) BioProject database under accession number PRJNA811393.
