# Supplementary material for: GM-CSF-miRNA-Jak2/Stat3 Signaling Mediates Chemotherapy-Induced Cancer Cell Stemness in Gastric Cancer
Source: Front Pharmacol. 2022 May 5;13:855351. doi: 10.3389/fphar.2022.855351 (PMC9117965; doi:10.3389/fphar.2022.855351)

Supplemental Figure Legends

Supplemental Figure S1. Validation of miR-877-3p overexpression in SGC7901 (A,B) and BGC823 (C,D) cells treated with 5-FU and DDP respectively. Data are presented as the mean ± SEM (N=3). *p<0.05, **p<0.01, ***p<0.001.

Supplemental Figure S2. Validation of miR-877-3p overexpression in SGC7901 (A) and BGC823 (B) cells. Data are presented as mean±SEM (N=3), *p<0.05, **p<0.01, ***p<0.001.

Supplemental Figure S3. Validation of miR-877-3p knockdown in SGC7901 (A) and BGC823 (B) cells. Data are presented as mean±SEM (N=3), *p<0.05, **p<0.01, ***p<0.001.

Supplemental Figure S4. Validation of SOCS2 downregulation at the mRNA levels in SGC7901 (A) and BGC823 (B) cells after overexpression of miR-877-3p. Data are presented as the mean ± SEM (N=3). *p<0.05, **p<0.01, ***p<0.001.

Supplemental Figure S5. Validation of SOCS2 upregulation at the mRNA levels in SGC7901 (C) and BGC823 (D) cells after knockdown of miR-877-3p. Data are presented as the mean ± SEM (N=3). *p<0.05, **p<0.01, ***p<0.001.

Supplemental Figure S6. miR-877-3p promoted gastric cancer cell proliferation. A and B: Overexpression of miR-877-3p in SGC7901 (A) and BGC823 (B) cells promoted cell proliferation assayed by CCK8. C and D: Overexpression of miR-877-3p in SGC7901 (C) and BGC823 (D) cells promoted the cellular colony formation. Data are presented as the mean ± SEM (N=3). *p<0.05, **p<0.01, ***p<0.001.

Supplemental Figure S7. miR-877-3p promoted gastric cancer cell stemness. A: Overexpression of miR-877-3p in SGC7901 and BGC823 cells promoted the proportion of $CD133^+$ CSCs. B and C: Overexpression of miR-877-3p in SGC7901 (B) and BGC823 (C) cells promoted the sphere formation ability in the serum-free culture condition. Data are presented as the mean ± SEM (N=3). *p<0.05, **p<0.01, ***p<0.001.

Supplemental Figure S1

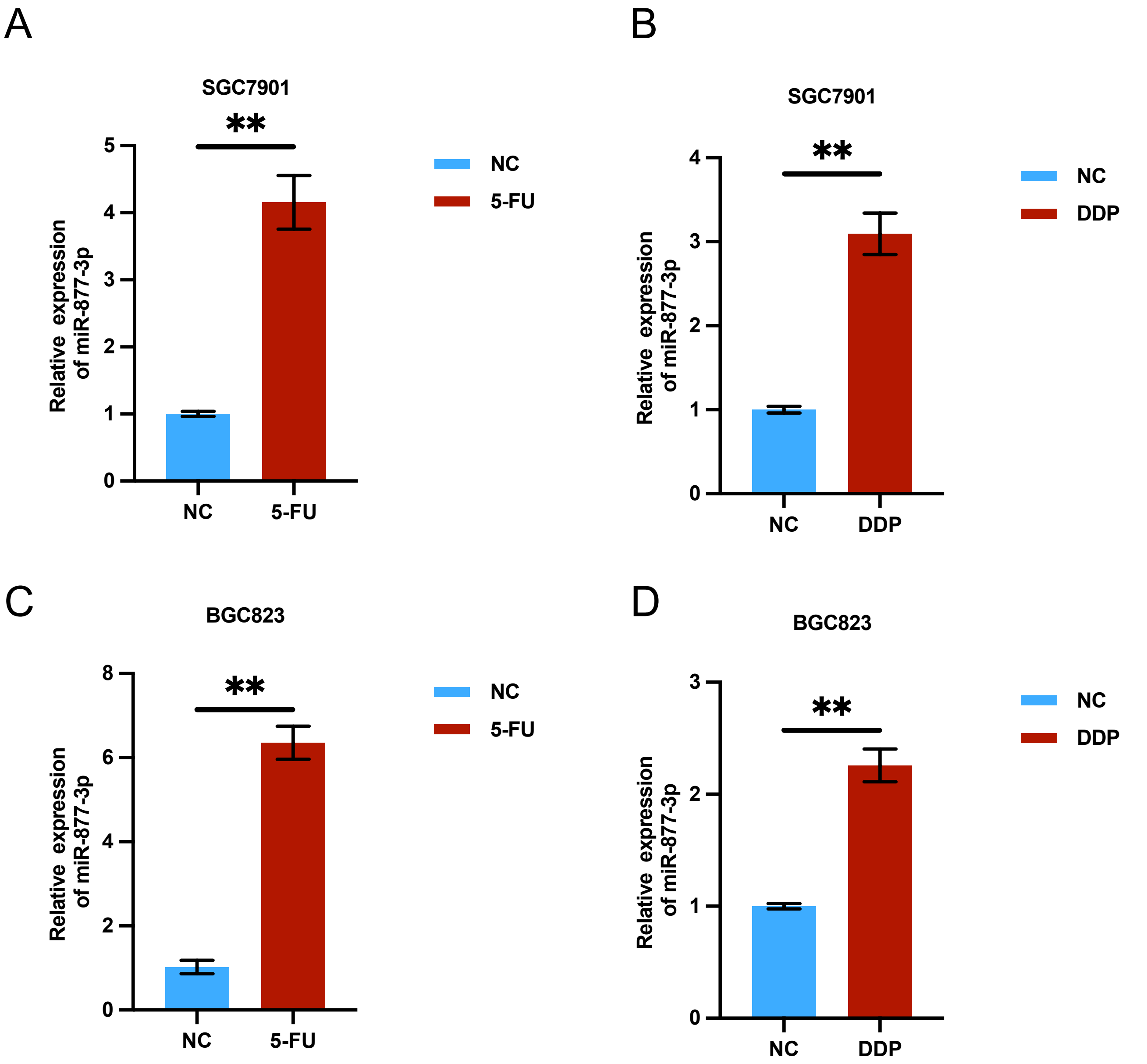

Supplemental Figure S2

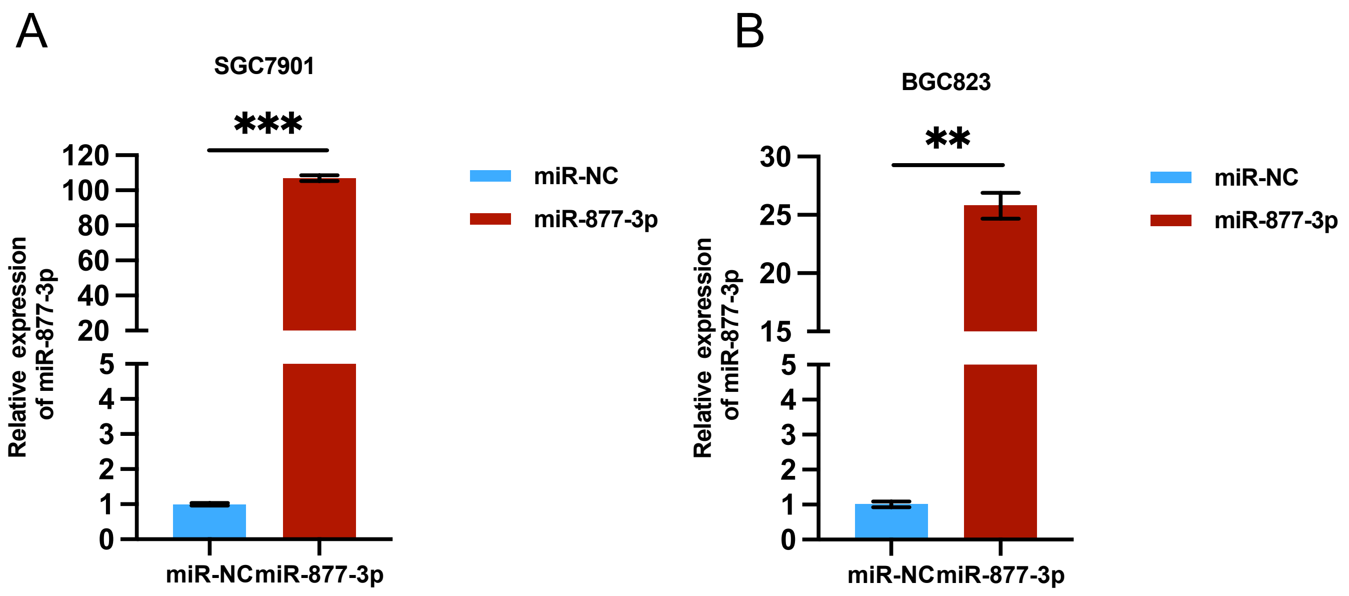

Supplemental Figure S3

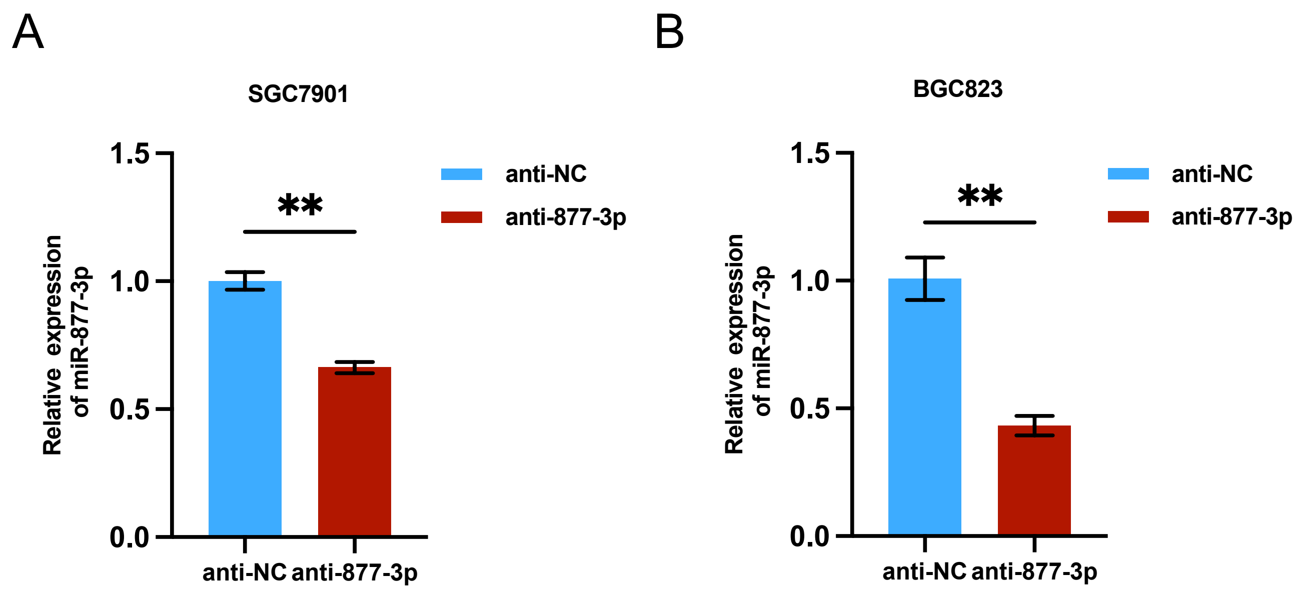

Supplemental Figure S4

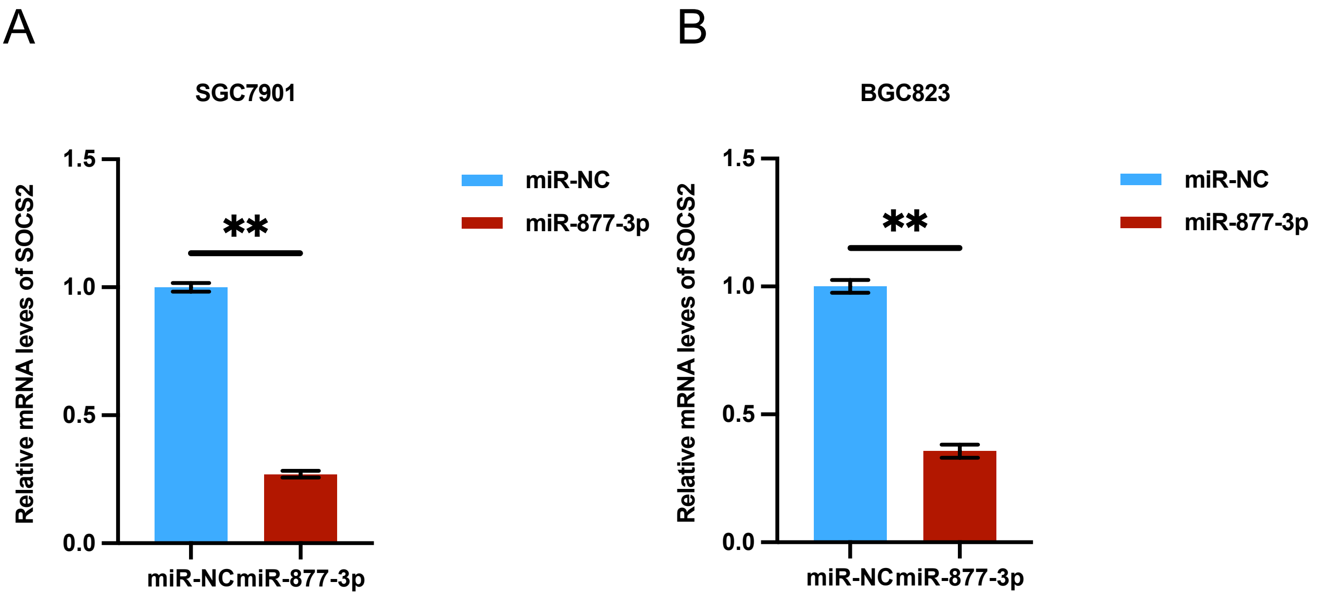

Supplemental Figure S5

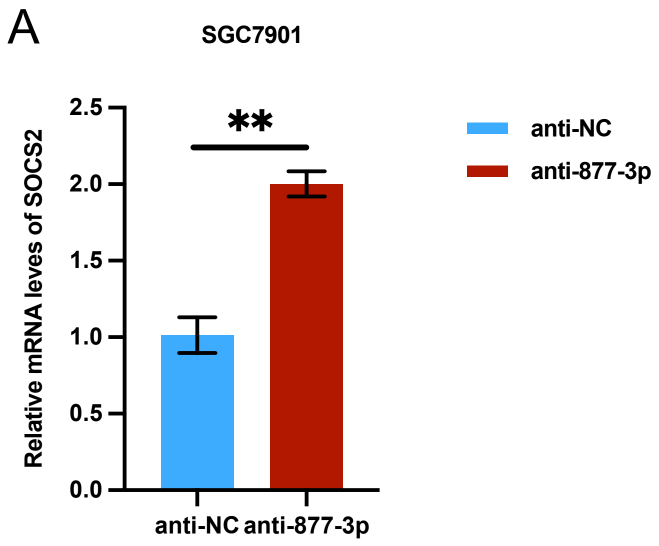

Supplemental Figure S6

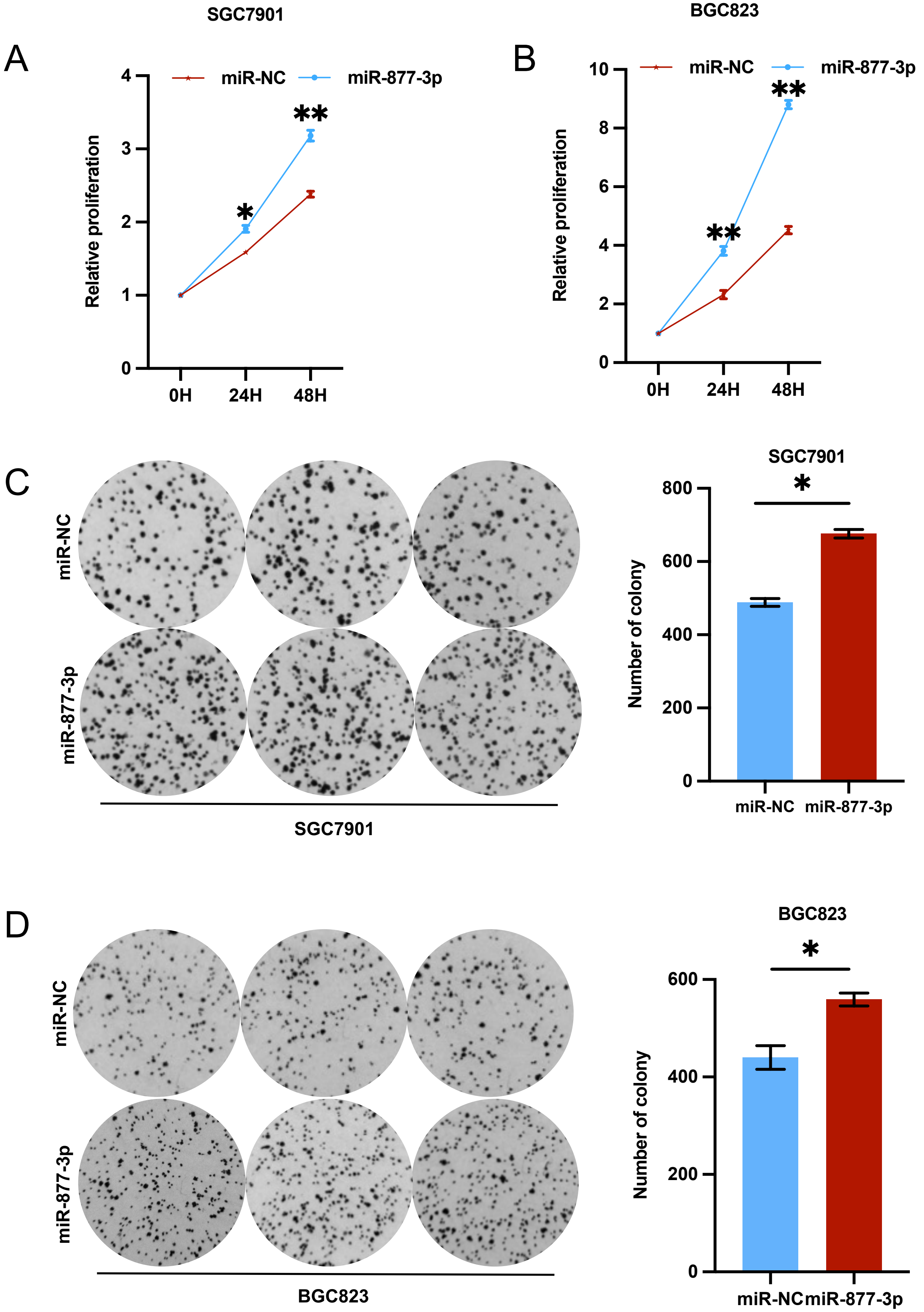

Supplemental Figure S7

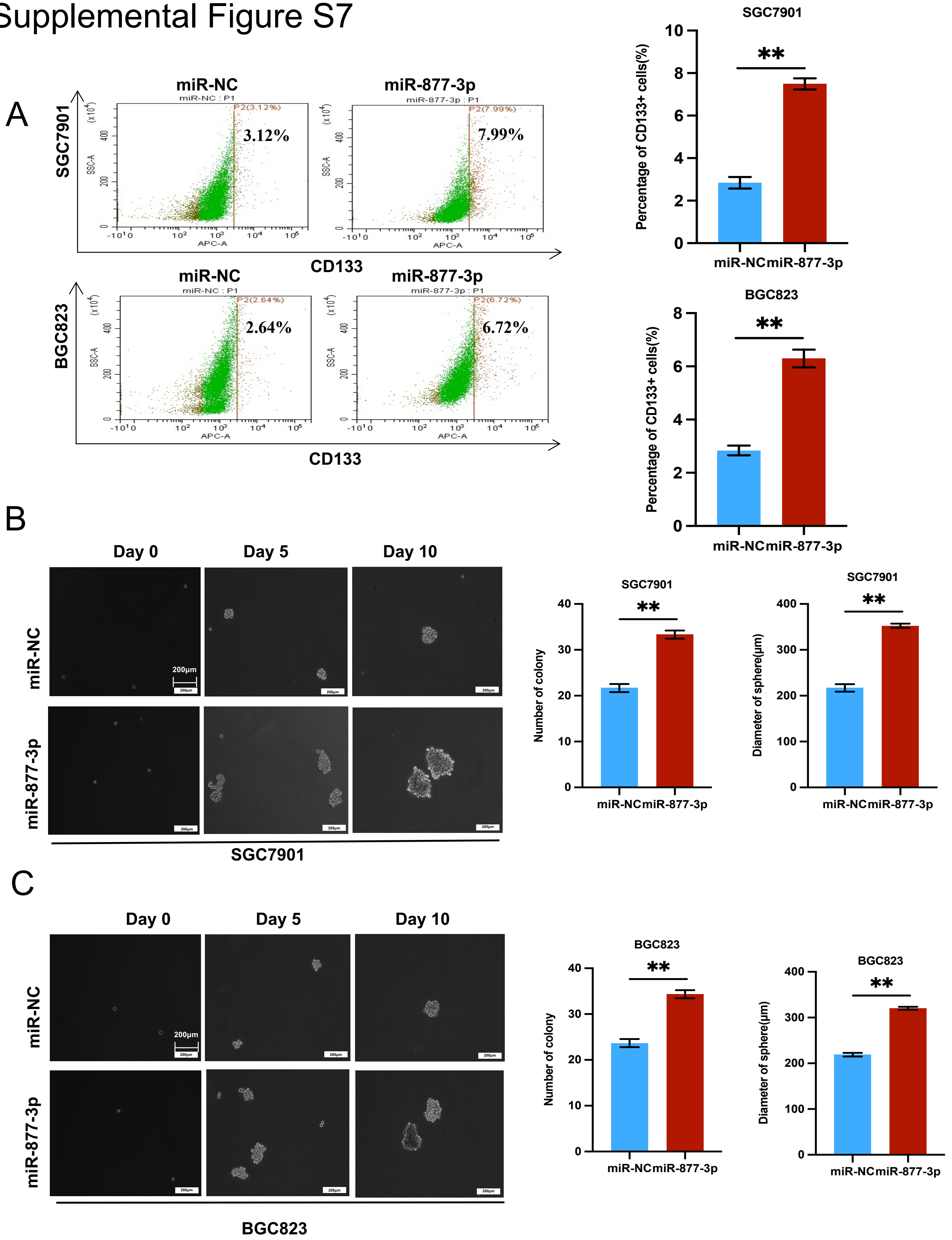

Supplement: Supplementary file 1 [file DataSheet1.pdf]
